# Mortality and cardiovascular disease burden of uncontrolled diabetes in a registry-based cohort: the ESCARVAL-risk study

**DOI:** 10.1186/s12872-018-0914-1

**Published:** 2018-09-04

**Authors:** Jorge Navarro-Pérez, Domingo Orozco-Beltran, Vicente Gil-Guillen, Vicente Pallares, Francisco Valls, Antonio Fernandez, Ana María Perez-Navarro, Carlos Sanchis, Alejandro Dominguez-Lucas, Jose M. Martin-Moreno, Josep Redon, Maria Tellez-Plaza, on behalf of the ESCARVAL STUDY GROUP

**Affiliations:** 1 Biomedical Research Institute INCLIVA, Hospital Clinico Universitario de Valencia, University of Valencia, Valencia, Spain; 2 Ciber of Epidemiology and Public Health (CIBERESP), Instituto de Salud Carlos III, Madrid, Spain; 3 Department of Clinical Medicine, University Miguel Hernandez of San Juan de Alicante, San Juan de Alicante, Spain; 4 Department of Medicine, University Jaume I of Castellón, Unión de Mutuas de Castellón, Castellón de la Plana, Spain; 5 Health Center of Beniganim, Valencia. HTA Working Group SEMERGEN, Valencia, Spain; 6 Escarval Project, Valencia, Spain; 7 Centro de Salud Algemesi, Algemesí, Spain; 8 Department of Preventive Medicine and Public Health, School of Medicine, University of Valencia, Valencia, Spain; 9 CIBERObn, Instituto de Salud Carlos III, Madrid, Spain; 10 Department of Environmental Health Sciences, Johns Hopkins Bloomberg School of Public Health, Baltimore, USA; 11 Hypertension Clinic, Clinical Hospital of Valencia, Avda Blasco Ibañez, 17, 46010 Valencia, Spain

**Keywords:** HbA1c, Diabetes, Attributable risk, All-cause mortality, Hospitalization, Coronary heart disease, Stroke

## Abstract

**Background:**

Despite the epidemiological evidence about the relationship between diabetes, mortality and cardiovascular disease, information about the population impact of uncontrolled diabetes is scarce. We aimed to estimate the attributable risk associated with HbA1c levels for all-cause mortality and cardiovascular hospitalization.

**Methods:**

Prospective study of subjects with diabetes mellitus using electronic health records from the universal public health system in the Valencian Community, Spain 2008–2012. We included 19,140 men and women aged 30 years or older with diabetes who underwent routine health examinations in primary care.

**Results:**

A total of 11,003 (57%) patients had uncontrolled diabetes defined as HbA1c ≥6.5%, and, among those, 5325 participants had HbA1c ≥7.5%. During an average follow-up time of 3.3 years, 499 deaths, 912 hospitalizations for coronary heart disease (CHD) and 786 hospitalizations for stroke were recorded. We observed a linear and increasingly positive dose-response of HbA1c levels and CHD hospitalization. The relative risk for all-cause mortality and CHD and stroke hospitalization comparing patients with and without uncontrolled diabetes was 1.29 (95 CI 1.08,1.55), 1.38 (95 CI 1.20,1.59) and 1.05 (95 CI 0.91, 1.21), respectively. The population attributable risk (PAR) associated with uncontrolled diabetes was 13.6% (95% CI; 4.0–23.9) for all-cause mortality, 17.9% (95% CI; 10.5–25.2) for CHD and 2.7% (95% CI; − 5.5-10.8) for stroke hospitalization.

**Conclusions:**

In a large general-practice cohort of patients with diabetes, uncontrolled glucose levels were associated with a substantial mortality and cardiovascular disease burden.

## Introduction

Prolonged exposure to hyperglycemia results in vascular damage [1]. The association between chronic hyperglycemia and cardiovascular complications, however, is not fully understood [2]. The HbA1c level is an indicator of the average blood glucose concentrations over the preceding 2–3 months and is used as a biomarker of diabetes control in clinical practice [3]. There is much evidence on the role of hyperglycemia as a cardiovascular disease (CVD) risk factor, sudden death [4], mortality in myocardial infarction [5] and mortality in critically ill patients [6], despite the fact that elevated HbA1c has shown inconsistent risk stratification, according to different levels [7, 8]. Observational studies and meta-analyses report that patients with uncontrolled diabetes, defined as HbA1c > 6.5%, are at increased risk for CVD and mortality compared to patients with controlled diabetes [7, 8]. Moreover, post hoc analyses from the Action to Control Cardiovascular Risk in Diabetes (ACCORD) [9]; the Action in Diabetes and Vascular Disease: Preterax and Diamicron Modified Release Controlled Evaluation (ADVANCE) [10], and the Veterans Affairs Diabetes Trial (VADT) [11], suggested different effects of glycemic control in patients with and without previous vascular disease. Furthermore, the HbA1c level is an independent risk factor for CV events, regardless of the diabetes diagnosis [12–15].

Despite the large number of studies analysing the prognostic value of HbA1c levels, information about the population impact associated with uncontrolled diabetes is scarce. The fraction of mortality and CVD potentially avoidable by achieving certain HbA1c levels in the population of individuals with diabetes has not been evaluated, and can be estimated by using real world data from Electronic Health Recording (EHR) [16]. Moreover, EHR-based studies from general-practice settings may provide a privileged view of the burden of disease associated to uncontrolled diabetes in the whole population. The ESCARVAL-RISK study, based on HER, is a cohort of beneficiaries of the universal health care system of the Valencian Community, (Spain), with CV risk factors, including diabetes, considered by a network of general practice physicians [17–19]. The objective of the present study was to estimate the attributable risk of all-cause mortality and CV hospitalization associated to elevated HbA1c levels in a cohort of patients with diabetes mellitus from the ESCARVAL-RISK study.

## Materials and methods

### Study population and baseline data collection

The sample was recruited from beneficiaries of the Valencian Health Agency’s universal health care system. The Valencian Community is a Mediterranean region located on the East-coast of Spain, with a population of 3,205,724 people older than 30 years in 2007. Total population data was extracted using the health information exchange function of ABUCASIS for the period of time between 1st January 2008 and 31st December 2012. ABUCASIS includes information on patient demographics, medications, vital status, past medical history and laboratory data, among others. Detailed information about the ESCARVAL-RISK project and data collection methods has been previously published [17–19].

**Fig. 1:**
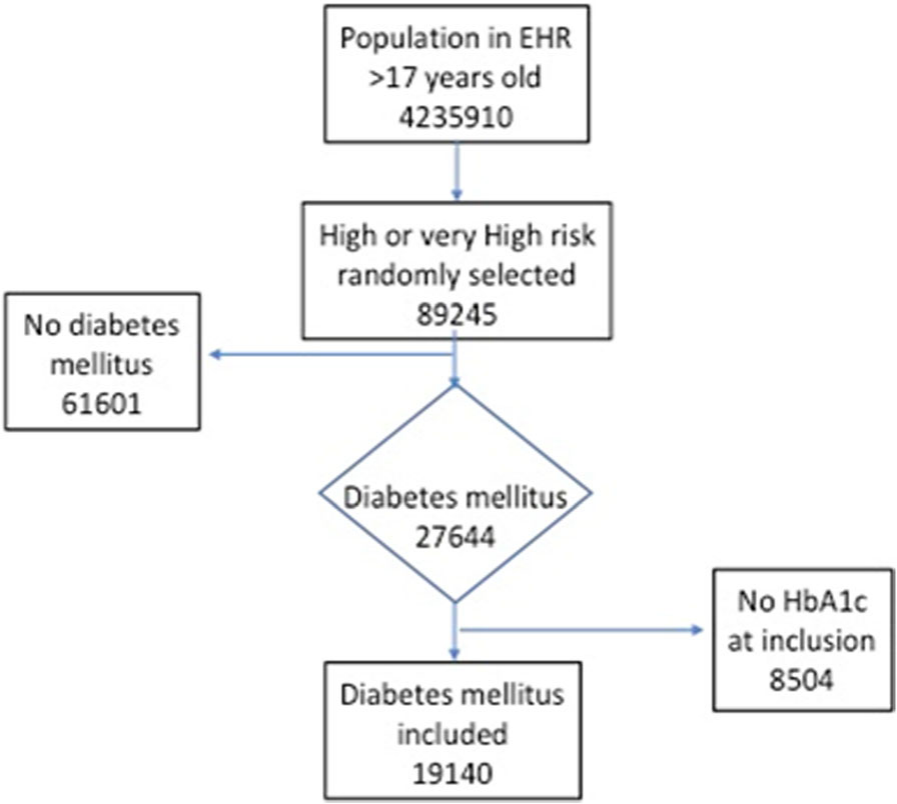
Flow-chart of the study population

Eligible patients for the present study were men and women with diabetes mellitus and free of CV disease. Participants were included in the study from 1st January 2008 if they fulfilled the eligibility conditions of a diagnosis of diabetes as a non-fasting glucose level of ≥200 mg/dl, a recorded physician diagnosis, use of glucose lowering drugs or insulin, or HbA1c ≥ 6.5%. Subsequently, participants newly diagnosed with these conditions during the study period were also included. The ESCARVAL-RISK is an observational study with the described previously characteristics [17–19]. Patient data was saved from various occasions and locations when they had received care, such as in the primary care physician’s office, as well as other physician specialists, nurses’ offices, pharmacies, hospitals, and emergency departments. As a result, not all the baseline variables that were needed to adjust for potential confounding in this study were available at the exact time of inclusion. We thus defined 6-month windows around the time of study inclusion in order to gather complete information on biochemistry results and blood pressure determinations) and excluded patient with missing dat. Finally, 19,140 diabetic subjects of both sexes aged 30 years or older who attended routine health examinations and fulfilled eligibility criteria were selected from the total population database. The STROBE chart is in Fig. 1.

### Cardiovascular risk factors definition

Body mass index (BMI) was calculated by dividing measured weight in kilograms by measured height in metres squared. Obesity was defined as a BMI ≥30 kg/m^2^. Blood pressure was measured up to three times on the same day in a sitting position and hypertension was defined as an office mean systolic blood pressure ≥ 140 mmHg, a mean diastolic blood pressure ≥ 90 mmHg, a recorded physician diagnosis, or antihypertensive medication use. Serum total cholesterol was measured enzymatically using the Cholesterol High Performance reagent (Roche Diagnostics). High density-lipoprotein (HDL) cholesterol was measured using a direct HDL reagent (Roche Diagnostics). Low density-lipoprotein LDL cholesterol was calculated by using the Friedwald formula, considering the triglyceride levels. High cholesterol was defined as a serum total cholesterol > 200 mg/dL, recorded diagnosis or medication use. HbA1c was assessed by the labs of different hospitals of the Valencia Community using High performance Liquid Chromatography (HPLC).

### Mortality and hospitalization follow-up

ESCARVAL-RISK participants were followed for all-cause mortality or the first episode of hospitalization for myocardial infarction or stroke until 31st December 2012. Causes of hospitalization were recorded using codes of the International Classification of Diseases, 9th Revision. Vital status was determined by matching ESCARVAL records and death certificates from the Spanish National Death Index. Mortality included all causes of death. Cause-specific hospitalization was defined as the first hospital admission for CHD (ICD-9 codes 410–414), and stroke (ICD-9 codes 430–438, 444). Time to event was calculated for each individual as the difference between the date of the inclusion into the study and the date of the hospital admission, the date of death, or 31st December 2012, whichever occurred first. A minimum follow-up period of one year was required.

### Confidentiality and ethics requirements

Patients’ data collected from the ABUCASIS system during the study were documented and pseudo-anonymized. The data generated during the study was handled according to Spanish Law 5/1999 and corresponding to European norms. The study was conducted according to the standards of the International Guidelines for Ethical Review of Epidemiological Studies (Council for International Organizations of Medical Sciences-CIOMS-Geneva, 1991) and the recommendations of the Spanish Society of Epidemiology about the review of ethical aspects of epidemiological research. The ESCARVAL-RISK study was reviewed and approved by the Committee for Ethics and Clinical Trials of the Center for Public Health Research (DGSP-CSISP).

### Statistical analysis

HbA1c levels were normally distributed and cut-offs for HbA1c categories and other quantiles used in the analysis were based on the distribution in the study sample. In descriptive analyses, we used generalised linear models to estimate means and proportions of participants’ characteristics, overall and by HbA1c categories. We also estimated, age and sex-adjusted rates for mortality and CV hospitalization end-points using Poisson regression for individual data with over-dispersion correction. Multi-adjusted rate differences were estimated from semi-parametric Aalen additive hazard models. To graphically display non-linear relationships, we used restricted quadratic splines with knots at the 20th, 50th and 80th percentiles of HbA1c distribution. Statistical models were adjusted for potential confounders.

Population attributable risks (PARs) for high HbA1c were calculated by using the standard formula PAR = 1 – Σ_*j*_Σ_*i*_ *p*_*ij*_ / *RR*_*i| j*_ [17, 18]. In this formula, the subscript *i* denotes one of two categories of HbA1c levels (with each participant classified as above or below the cut-off being used to calculate the PAR, respectively), the subscript *j* is an index for all strata obtained after cross-classifying the study sample for all adjusted covariates, *p*_*ij*_ is the proportion of cases over all cases in the study population in each stratum after cross-classifying the dichotomous HbA1c categories and all adjusted covariates, and *RR*_*i*|*j*_ is the adjusted hazard ratio for the endpoint of interest comparing participants below and above the HbA1c cut-off in stratum *j* of covariates, from Cox proportional hazards regression. We calculated adjusted PARs for HbA1c > 6.5% from separate models fully adjusted for age (restricted quadratic splines with 5 knots), sex, smoking status (never, former, current), obesity (no, yes), hypertension (no, yes), chronic kidney disease (no, yes), anti-hypertensive medication (no, yes), glucose lowering medication (no, yes), lipid lowering medication (no, yes).

## Results

### Participant characteristics

The mean age was 65.1 years and 54.3% were males. At baseline, 49.6% participants were obese (average BMI 30.4 kg/m2), 83.2% participants had hypertension and 16.7% participants showed total cholesterol > 200 mg/dl, (among those, 38% were receiving lipid lowering drugs). 11,003 (57%) patients had uncontrolled diabetes defined as HbA1c ≥6.5%, and, among those, 5325 participants had HbA1c ≥7.5%. Patients were treated with insulin, 8.3%, and 43.5% with different oral glucose lowering agents. The main characteristics of the study population by HbA1c levels are shown in Table 1. Participants with increasing HbA1c levels showed increasing current smoking and glucose lowering medication status and decreasing HDL cholesterol levels.

**Table 1:** Baseline Characteristics of Study Participants Overall and by Glycated Hemoglobine categories

	Overall (*N* = 19,140)	< 6 (*N* = 4304)	6–6.5 (*N* = 3773)	6.5–7 (*N* = 3339)	7–7.5 (*N* = 2399)	> = 7.5 (*N* = 5325)	p-trend
Age, years; mean	65.1 (0.1)	65.4 (0.2)	66.2 (0.2)	66 (0.2)	66.2 (0.2)	62.9 (0.2)	< 0.001
Men; %	54.3 (0.4)	54.8 (0.8)	50 (0.8)	51.4 (0.9)	53.3 (1)	59 (0.7)	< 0.001
Obesity; %	49.6 (0.4)	47.4 (0.8)	51.5 (0.8)	49.3 (0.9)	49.7 (1)	50.1 (0.7)	0.098
BMI, kg/m^2^; mean	30.4 (0)	30.2 (0.1)	30.6 (0.1)	30.4 (0.1)	30.5 (0.1)	30.5 (0.1)	0.05
Former smoking; %	23.4 (0.3)	24 (0.7)	23.2 (0.7)	22.6 (0.7)	22.9 (0.9)	23.8 (0.6)	0.875
Current smoking; %	21.6 (0.3)	18.5 (0.6)	18.4 (0.6)	20.1 (0.7)	20.5 (0.8)	27.8 (0.6)	< 0.001
Glucose lowering medication; %	51.8 (0.4)	38.8 (0.7)	45.6 (0.8)	52 (0.9)	60 (1)	62.8 (0.7)	< 0.001
Hypertension; %	83.2 (0.3)	83.8 (0.6)	84.1 (0.6)	84.4 (0.6)	83.6 (0.8)	81 (0.5)	< 0.001
Antihypertensive medication; %	49 (0.4)	50.4 (0.8)	50.2 (0.8)	52.4 (0.9)	50.3 (1)	44.3 (0.7)	< 0.001
Systolic blood pressure, mmHg; mean	138 (0.1)	136.1 (0.3)	137 (0.3)	138.3 (0.3)	138.8 (0.4)	139.9 (0.3)	< 0.001
Diastolic blood pressure, mmHg; mean	78.6 (0.1)	78 (0.2)	78.3 (0.2)	78.7 (0.2)	78.3 (0.2)	79.4 (0.1)	< 0.001
High cholesterol; %	16.7 (0.3)	18.1 (0.6)	17.2 (0.6)	16.8 (0.7)	16.8 (0.8)	15 (0.5)	< 0.001
Total cholesterol, mg/dL; mean	197.5 (0.3)	195.5 (0.6)	197 (0.7)	196.3 (0.7)	194.3 (0.8)	201.8 (0.6)	< 0.001
HDL-cholesterol, mg/dL; mean	48.9 (0.1)	50.3 (0.2)	49.9 (0.2)	49.4 (0.2)	48.6 (0.3)	46.8 (0.2)	< 0.001
LDL-cholesterol, mg/dL; mean	116.6 (0.2)	116.1 (0.5)	116.3 (0.5)	115.3 (0.6)	113.7 (0.7)	119.3 (0.5)	< 0.001
Lipid lowering medication; %	38 (0.3)	35.6 (0.7)	39.2 (0.8)	40.1 (0.9)	40.7 (1)	36.6 (0.7)	0.458

### Dose-response associations

**Fig. 2:**
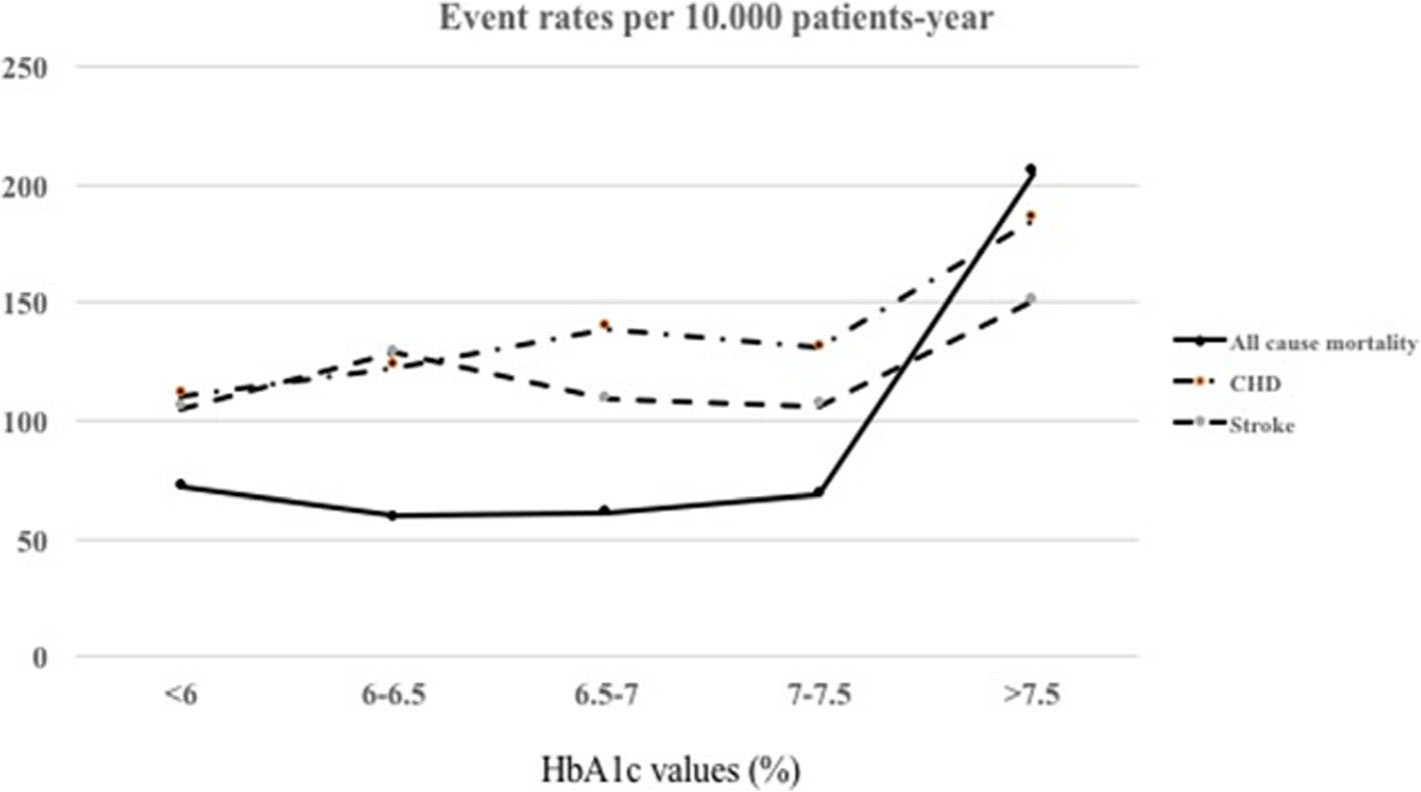
Age and Sex-adjusted rates (events/10,000 person-years) of all-cause mortality and CVD hospitalization by quartile of HbA1c

**Fig. 3:**
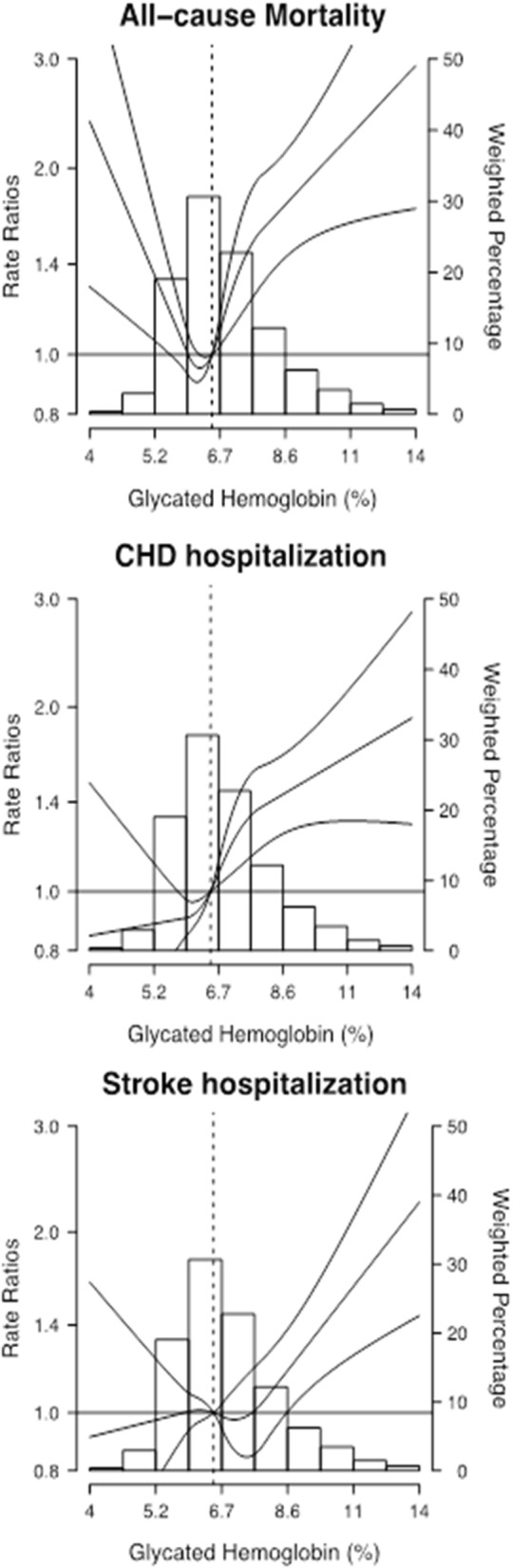
Adjusted Odds Ratios (95% CI) of Mortality and morbidity by Glycated Hemoglobin levels The curve represents adjusted rate ratio (RR) of mortality (panel 1), CHD hospitalization (panel 2) and stroke hospitalization (panel 3) by glycated hemoglobin levels, based on restricted quadratic splines with knots at the 10th, 50th, and 90th percentiles (5.6%, 6.7% and 8.9%, respectively) of the glycated hemoglobin distribution. The reference value (RR = 1) was set at 5.6% of the Glycated Hemoglobin. RRs were adjusted for age (restricted quadratic splines with 5 knots), sex, smoking status (never, former, current), obesity (no, yes), hypertension (no, yes), chronic kidney disease (no, yes), HDL cholesterol ≤40 for men and ≤ 50 for women (no, yes), LDL ≥ 130 mg/dL (no, yes), anti-hypertensive medication (no, yes), glucose lowering medication (no, yes), lipid lowering medication (no, yes). For a given value (%) of glycated hemoglobin distribution, the corresponding RR is interpreted as the expected change in the rate of mortality and CVD hospitalization, associated to changing glycated hemoglobin from a given value, to the reference (6.5%). For example, model estimates suggest that the RR for mortality of participants in the 90th percentile of glycated hemoglobin (8.9%) is 80% higher, compared to participants in the Reference (6.5%).

During an average follow-up of 3.3 years, the EHR recorded 499 deaths, 912 hospitalizations for CHD and 786 hospitalizations for stroke. The age and sex-adjusted rates and events/10,000 person-years for all-cause mortality and CVD hospitalization by HbA1c levels are shown in Table 2 and Fig. 2. We observed a fairly linear and increasingly positive dose-response of CHD hospitalization by HbA1c levels (Table 2, Fig. 3, panel 2). For all-cause mortality, however, the dose-response by HbA1c levels was non-linear (Fig. 3, panel 1). For stroke hospitalization, the dose response was statistically significant only in the higher range of HbA1c distribution (Fig. 3, panel 3).

**Table 2:** Age and Sex-adjusted rates (events/10,000 person-years) of all-cause mortality and CVD hospitalization by quartile of HbA1c

	Glycated Hemoglobin categories					*p*-value
	< 6 (N = 4304)	6–6.5 (N = 3773)	6.5–7 (N = 3339)	7–7.5 (N = 2399)	> = 7.5 (N = 5325)	
Glycated Hemoglobin						
Median (range), mg/dL	5.6 (4.6, 5.9)	5.6 (6, 6.4)	6.7 (6.5, 6.9)	7.2 (7, 7.4)	8.4 (7.5, 12.6)	
All-cause mortality						
Cases (person-year)	116 (15,346.42)	80 (12,801.36)	72 (11,233.22)	60 (8174.11)	171 (17,893.89)	
Rate	72.1	59.6	60.7	68.8	104.8	< 0.001
CHD hospitalization						
Cases (person-year)	169 (14,979.12)	154 (12,477.02)	155 (10,951.90)	123 (7936.55)	311 (17,329.30)	
Rate	110.8	122.6	139.0	150.8	185.3	< 0.001
Stroke hospitalization						
Cases (person-year)	161 (15,025.62)	166 (12,480.03)	126 (11,002.67)	90 (8016.53)	243 (17,442.46)	
Rate	104.9	128.6	109.1	106.3	150.6	0.004

### Burden of disease associated to HbA1c level

In additive hazard models, the absolute risk differences for CHD hospitalization also progressively increased from 11.41 events/10,000 person-years for HbA1c levels between 6 and 6.5%, to 70.26 events/10,000 person-years for HbA1c levels greater than 7% (Table 3). The relative risk for all-cause mortality and CHD and stroke hospitalization comparing patients with and without uncontrolled diabetes was 1.29 (95 CI 1.08,1.55), 1.38 (95 CI 1.20,1.59) and 1.05 (95 CI 0.91, 1.21), respectively. The corresponding population attributable risk (PAR) associated with uncontrolled diabetes was 13.6% (95% CI; 4.0–23.9) for all-cause mortality, 17.9% (95% CI; 10.5–25.2) for CHD and 2.7% (95% CI; − 5.5-10.8) for stroke hospitalization (Table 4).

**Table 3:** Rate Differences (events/10.000 person-years [95% confidence interval]) for all-cause mortality and CVD endpoints after a 5-year follow-up by HbA1c categories

	C / NC	Total mortality		C / NC	CHD hospitalization		C / NC	Stroke hospitalization	
eGFR		Model 1	Model 2		Model 1	Model 2		Model 1	Model 2
< 6	116 / 4188	Ref.	Ref.	169 / 4135	Ref.	Ref.	161 / 4143	Ref.	Ref.
6–6.5	80 / 3693	−13.26 (−32.40, 5.88)	−11.44 (−30.75, 7.86)	154 / 3619	11.71 (−13.33, 36.74)	11.41 (−13.57, 36.39)	166 / 3607	24.14 (−1.22, 49.50)	22.30 (−3.16, 47.77)
6.5–7	72 / 3267	− 12.42 (− 32.08, 7.23)	−6.93 (−26.87, 13.02)	155 / 3184	28.06 (−0.06, 56.17)	25.04 (− 3.36, 53.45)	126 / 3213	3.64 (−22.45, 29.74)	0.10 (−26.13, 26.32)
7–7.5	60 / 2339	−5.34 (−27.81, 17.13)	4.24 (−18.73, 27.20)	123 / 2276	39.60 (7.54, 71.66)	37.34 (4.92, 69.76)	90 / 2309	− 0.06 (− 27.92, 27.81)	−4.88 (− 33.34, 23.57)
> = 7	171 / 5154	28.26 (9.17, 47.36)	34.22 (14.55, 53.90)	311 / 5014	72.24 (46.83, 97.64)	70.26 (44.43, 96.09)	243 / 5082	42.74 (18.58, 66.90)	35.15 (10.51, 59.79)
P-trend		0.001	< 0.001		< 0.001	< 0.001		0.007	0.044

Model 1 is adjusted for age and sex. Model 2 is further adjusted smoking status (never, former, current), obesity (no, yes), hypertension (no, yes), chronic kidney disease (no, yes), HDL cholesterol ≤40 for men and ≤ 50 for women (no, yes), LDL ≥ 130 mg/dL (no, yes), anti-hypertensive medication (no, yes), glucose lowering medication (no, yes), lipid lowering medication (no, yes)

**Table 4:** Population attributable risk of all-cause mortality and CVD hospitalization, associated to HbA1c > 6.5%

	All-cause mortality	CHD hospitalization	Stroke hospitalization
Cases/Non-cases	499/18,598	912/18,185	786/18,311
RR	1.29 (1.08, 1.55)	1.38 (1.2, 1.59)	1.05 (0.91, 1.21)
PAR %	13.62 (3.96, 22.90)	17.90 (10.53, 25.20)	2.64 (−5.51, 10.76)

Model is fully adjusted for age (restricted quadratic splines with 5 knots), sex, smoking status (never, former, current), obesity (no, yes), hypertension (no, yes), chronic kidney disease (no, yes), anti-hypertensive medication (no, yes), glucose lowering medication (no, yes), lipid lowering medication (no, yes). The prevalence of glycated hemoglobin > 6.5% was 57.78%

## Discussion

In a large cohort of individuals with diabetes from a Mediterranean region in Spain, glucose control assessed by HbA1c levels was positively related to the risk of all-cause mortality, as well as hospitalization by CHD, starting with HbA1c levels greater than ~ 6%. The association with stroke hospitalization, however, was only significant in the higher range of HbA1c levels. The fraction of potentially avoidable deaths associated with having HbA1c below 6.5% was 14%. The corresponding fraction of avoidable CHD hospitalizations was 18% (Table 5).

**Table 5:** Main messages on the mortality and cardiovascular disease burden of uncontrolled diabetes in a registry-based cohort: the ESCARVAL-risk study

Main messages There is much evidence on the role of hyperglycemia as a cardiovascular disease The HbA1c level is used as a biomarker of diabetes control in clinical practice and is an independent risk factor for CV events, regardless of the diabetes diagnosis Information about the population impact associated with uncontrolled diabetes is scarce In a large cohort of individuals with diabetes, HbA1c levels was positively related to the risk of all-cause mortality, as well as hospitalization by CHD, starting with HbA1c levels > 6%. The fraction of potentially avoidable deaths associated with having HbA1c below 6.5% was 14% and the corresponding fraction of avoidable CHD hospitalizations was 18%.	

The present study was conducted in a population with a country-specific low CV risk profile as referenced by the SCORE study [20], even though the prevalence of diabetes is 13.8% [21]. In the whole Valencian Community territory, the EHR associated with the public general-practice setting has a 92% coverage of the population living in the area [22]. Every patient has a unique personal identification number which guarantees the interoperability of the EHRs. Thus, administrative data, including all prescriptions and dispensation of subsidized treatments for diabetes and hospitalization events are linked to the database that integrates all the health care interventions and procedures that the patients received. Therefore, this study includes information on baseline risk factors and follow–up for mortality and CV hospitalizations from essentially all adults with diabetes mellitus in the region who had their HAb1a levels measured by the public health system during the study period.

The present study supports that the risk of death and CHD hospitalization is related to glucose-control and points to the substantial potential for prevention associated to lowering HbA1c levels. Despite the epidemiological evidence about the relationship between glucose control, assessed by HbA1c, and CV events, randomized clinical trials did not show a benefit of better glucose-control in reducing risk. Thus, evidence was not sufficient to generate strong recommendations for clinical practice. In fact, the evidence was graded IIa/C in the ACCF/AHA guidelines for assessment of CV risk in asymptomatic adults [23] and by the ESC-EARD [24]. Recent revisions of guidelines from Scientific Societies moved away from uniform recommendations and towards a more nuanced patient-centered approach to HbA1c therapeutic targets [25]. It is possible that a minimum study duration and a minimum gain in HbA1c reduction are necessary to drive a relevant risk reduction in CV risk [26]. In the absence of conclusive evidence from RCTs, observational epidemiological studies using real world data [27] might provide additional useful information to clarify the attributable risk of glucose control in CV risk.

The population impact of uncontrolled diabetes in the present study is displayed in three ways. First, we show age and sex-adjusted absolute rates for mortality or hospitalization for CHD or stroke by HbA1c subgroups of interest. The rate of CHD hospitalization shows a progressive increment from the lowest to the highest level, while the rate of stroke hospitalization is less constant across the HbA1c categories. In agreement with the present study, a meta-analysis showed that 1% HbA1c reduction was associated with a lowered major CV risk by glycemic control, but was not associated with lowered stroke and death risks [7]. Studies with strong association with stroke come from Taiwan [28] and Korea [8], in which the risk of stroke is high compared with Caucasians. For all-cause mortality, but not for CHD or stroke hospitalization, a U-shape curve was observed, such as that in the UK GPRD and ARIC, studies that showed increased risk of all-cause death with both lower and higher HbA1c levels [13, 29]. However, the U-shaped curve was not confirmed in other studies such as the Swedish National Diabetes Register cohort [30].

Second, we calculated the multi-adjusted rate differences in mortality or hospitalizations (also termed “attributable risk”), comparing patients with progressively increasing HbA1c levels to HbA1c levels lower than 6%. This can be interpreted as the average annual increase in mortality and CV disease hospitalization risk on an absolute scale attributable to HbA1c categories adjusting for potential confounders. In both all-cause mortality and stroke hospitalization the attributable risks were only statistically significant at HbA1c > 7.5%. For CHD hospitalization, however, the attributable risk became borderline statistically significant at the 6.5% cut-off, but displayed a progressive increase across HbA1c categories. Moreover, at the higher range of HbA1c levels, the attributable risk for CHD hospitalization doubles. These results have not been previously reported in other cohorts of patients with diabetes, but support the current target levels of HbA1c < 7% recommended by the guidelines of the American Diabetes Association [31].

Third, we estimated the adjusted PAR associated to uncontrolled diabetes defined as HbA1c > 6.5%, which represents the estimated fraction of deaths that would be avoided in the population. This assumes that the effects are causal and that other risk factors remained unchanged, and therefore suggests that strict diabetes control could relevantly diminish the CV disease burden of diabetes. More studies are needed to confirm the findings.

The study needs to be considered within its strengths and limitations. The main limitation of the present study is the lack of information about the duration and progression of the CV risk factors. Thus, some degree of residual confounding cannot be discarded. Another limitation is the absence of HbA1c follow-up data; therefore, it was not possible to test the potential role of HbA1c individual trajectories on mortality and CV risk. In addition, the ascertainment of CHD and stroke hospitalizations was performed mainly through hospital discharge codes, which may have led to the under-ascertainment of cases that perhaps were not severe enough to warrant hospitalization. Finally, it is possible that findings from this Mediterranean cohort are not generalizable to other populations. However, the EHR system of the Valencian Community (ABUCASIS), which is the framework for the ESCARVAL project, allowed the monitoring of a population-based sample of individuals with diabetes throughout their experience in a general practice system. While the follow-up time in this study was not long, the large sample size, however, provided enough power and a valuable frame to assess the attributable risk of mortality and CHD and stroke associated to diabetes control in the short term. This provides a common scale for comparing the potential population-level impact of interventions for disease prevention.

## Conclusions

In conclusion, in a large general-practice cohort of patients with diabetes, uncontrolled diabetes was associated with a substantial mortality and CV disease burden. While our results support a potential benefit of decreasing HbA1c levels below the traditional 6.5% threshold in patients with diabetes, additional studies are needed to confirm these findings.

## Abbreviations

ACCF/AHA: American College of Cardiology/American Heart Association
ARIC: Atherosclerosis Risk in Communities
BMI: Body Mass Index
CHD: Hospitalizations for Coronary Heart Disease
CI: Confidence Interval
CV: Cardiovascular
CVD: Cardiovascular Disease
EHR: Electronic Health Recording
ESC-EARD: European Society of Cardiology - European Association for the Study of Diabetes
HbA1c: Glycated Haemoglobin
HDL: High Density Lipoprotein
HPLC: High Performance Liquid Chromatography
LDL: Low Density Lipoprotein
PAR: Population Attributable Risk
RCTs: Randomized Clinical Trials
UK GPRD: United Kingdom General Practice Research Database
